# Demonstration of *N*,*N*-Dimethyldithiocarbamate as a Copper-Dependent Antibiotic against Multiple Upper Respiratory Tract Pathogens

**DOI:** 10.1128/Spectrum.00778-21

**Published:** 2021-09-01

**Authors:** Sanjay V. Menghani, Angela Rivera, Miranda Neubert, James R. Hagerty, Lourdes Lewis, John N. Galgiani, Emmitt R. Jolly, Joseph W. Alvin, Michael D. L. Johnson

**Affiliations:** a Department of Immunobiology, University of Arizona, Tucson, Arizona, USA; b Department of Chemistry and Biochemistry, University of Arizona, Tucson, Arizona, USA; c Department of Biology, Case Western Reserve University, Cleveland, Ohio, USA; d Valley Fever Center for Excellence and Department of Medicine, University of Arizona, Tucson, Arizona, USA; e BIO5 Institute, University of Arizona, Tucson, Arizona, USA; f Center for Global Health and Disease, Case Western Reserve University, Cleveland, Ohio, USA; University of Georgia

**Keywords:** *Schistosoma*, *Staphylococcus aureus*, *Streptococcus pneumoniae*, Valley Fever, antibiotic, *Coccidioides*, copper-dependent toxicity

## Abstract

Transition metals are necessary cofactors and structural elements in living systems. Exposure to high concentrations of biologically important transition metals, such as zinc and copper, results in cell toxicity. At the infection site, the immune system deploys metal sorbent proteins (e.g., lactoferrin and calprotectin) to starve pathogens of necessary metals (such as iron), while phagocytes expose engulfed pathogens to high levels of other metals, such as copper and zinc. The opportunistic pathogen Streptococcus pneumoniae (the pneumococcus) encounters macrophages during initial and protracted infections. The pneumococcus employs a copper export pathway, which improves colonization and persistent infection of the nasopharynx and the upper respiratory tract. Because copper is tightly regulated in the host, we instead sought to leverage the localized power of nutritional immunity by identifying small molecules with copper-dependent toxicity (CDT) through a targeted screen of compounds for antibiotic efficacy. We chose to include dithiocarbamates, based on the copper synergy observed in other organisms with 1-(diethylthiocarbamoyldisulfanyl)-*N*,*N*-diethyl-methanethioamide (tetraethylthiuram disulfide, disulfiram). We observed CDT of some dithiocarbamates in S. pneumoniae. Only *N*,*N*-dimethyldithiocarbamate (DMDC) was consistently toxic across a range of concentrations with copper both *in vitro* and *in vivo* against the pneumococcus. We also observed various degrees of CDT *in vitro* using DMDC in Staphylococcus aureus, Coccidioides posadasii, and Schistosoma mansoni. Collectively, we demonstrate that the compound DMDC is a potent bactericidal compound against S. pneumoniae with antimicrobial efficacy against bacterial and fungal pathogens.

**IMPORTANCE** With the rise of antibiotic resistance, approaches that add new antimicrobials to the current repertoire are vital. Here, we investigate putative and known copper ionophores in an attempt to intoxicate bacteria and use ionophore/copper synergy, and we ultimately find success with *N*,*N*-dimethyldithiocarbamate (DMDC). We show that DMDC has *in vitro* efficacy in a copper-dependent manner and kills pathogens across three different kingdoms, Streptococcus pneumoniae, *Coccidioides posadasii*, and Schistosoma mansoni, and *in vivo* efficacy against S. pneumoniae. As such, dithiocarbamates represent a new potential class of antimicrobials and thus warrant further mechanistic investigation.

## INTRODUCTION

Microbial resistance to traditional antibiotics is an existential risk and a central focus of global health. Innovation tends to focus on well-studied, canonical targets, such as the cell wall (β-lactams) or translation (aminoglycosides) (1–3). This strong and near-global selection pressure is evident by examples of clinical resistance only years after introduction. By iteratively selecting a single target or cell function, a pathogen may only need a few mutations to escape. Conversely, the aseptic properties of copper are not completely understood, yet copper toxicity disrupts multiple components of cellular homeostasis. The current model for soluble copper toxicity *in vivo* is based on its Fenton-like redox activity and displacing native metal cofactors (4, 5). Within aerobic environments, it is thought that Cu^2+^ is the predominant ion species. Endogenous reducing agents such as glutathione are easily oxidized by Cu^2+^, affording the insoluble and reactive Cu^1+^ ion, and act to buffer excess copper (6, 7). Much like Fe^2+^, Cu^1+^ catalytically reduces H_2_O_2_ to hydroxide ions and hydroxyl radicals. Through these reactions, exposure to Cu^2+^ can lead to rapid generation of reactive oxygen species (ROS) and depletion of cellular antioxidants.

Streptococcus pneumoniae (the pneumococcus) is a causative agent of pneumonia, otitis media, meningitis, and sepsis. When grown aerobically, the pneumococcus uses pyruvate oxidase to generate acetyl phosphate, which also produces H_2_O_2_. S. pneumoniae does not produce a catalase, which might suggest that this bacterium is more sensitive to H_2_O_2_ stress. However, S. pneumoniae survives exposure to 10 mM H_2_O_2_ and produces large amounts of peroxide (∼100 μM h^−1^; [H_2_O_2_]_max_ of >1 mM) (8–12). Considering these conditions, S. pneumoniae is remarkably resistant to Cu^2+^ in standard media, overcoming concentrations above 2 mM (9, 10, 13). This resistance and the importance of copper export in pneumococcal colonization and persistence make this organism an appealing model to study aspects of copper toxicity.

The stability of transition metal complexes is generally understood through the Irving-Williams series: Mn^2+^ < Fe^2+^ < Co^2+^ < Ni^2+^ < Cu^2+^ > Zn^2+^ (14). Because of the extreme stability of Cu complexes, “free” copper ions are exceedingly rare. As such, copper ions can displace most native metals from essential proteins, commonly called mismetallation (5, 15, 16). Mismetallated proteins are catalytically poisoned, denatured, or otherwise inactivated. Host macrophages import Cu^1+^ into the oxidizing phagolysosome via ATP7A, resulting in Cu^2+^ toxicity against the phagocytosed organism (17). Indeed, in S. pneumoniae, Cu^2+^ displaces the Mn^2+^ cofactor of the aerobic ribonucleotide reductase (NrdF), starving the cell of deoxynucleotides and iron-sulfur clusters in Escherichia coli (16, 18).

While Cu itself is toxic, 8-hydroxyquinoline (8HQ) and related chelators can function as ionophores, overwhelming cells with Cu (19–25). FDA-approved drugs such as disulfiram also show potent copper-dependent toxicity (CDT) (24, 26–31). In this study, we report the effects of several small molecules’ activities on the growth and viability of S. pneumoniae in the presence or absence of copper. These compounds were tested using apparent population density (optical density at 600 nm [OD_600_]) and viable CFU as measures of antimicrobial activity. S. pneumoniae cultures were either grown overnight in various treatments or a growing culture was subjected to similar treatments. The overnight growth curve and CFU counting both served to determine if (i) the treatment would prevent bacterial growth from a small inoculum and if (ii) the treatment would kill an established population of bacteria (killing curves). In this manner, we validated whether a small molecule was a promising ‘hit’ for CDT.

Human copper levels vary widely with variables of cell type, genotype, and time plus a multitude of environmental factors. In the case of mammalian macrophages, synchrotron X-ray fluorescence (XF) was used to estimate intraphagosomal concentrations of Mycobacterium tuberculosis-containing vesicles. This XF study found that copper concentration within M. tuberculosis phagosomes widely varies (*μ* = 426 ± 393 μM; *n* = 7) after the first hour of infection (32). While broad (30 to 800 μM Cu), the upper limit is roughly 10- to 20-fold higher than a bacterium encounters outside the phagosome (33). Based on these studies, we argue that the concentrations used in this study (240 nM to 1 mM) are appropriate for the *in vitro* experiments below.

Here, we found that 8HQ + Cu^2+^ in S. pneumoniae had limited biocidal effects. We also found that disulfiram had no bactericidal properties on the pneumococcus in a metal-dependent manner. However, the primary metabolite of disulfiram, *N*,*N*-diethyldithiocarbamate (DETDC), demonstrated potent CDT at mid- to low-micromolar concentrations (<100 μM) but had no effect with copper at high concentrations. The related compound *N*,*N*-dimethyldithiocarbamate (DMDC) is currently used as a fungicide in complex with zinc as ziram (34). DMDC displayed CDT at even lower tested concentrations than DETDC against the pneumococcus. We performed further *in vivo* testing of DMDC against the pneumococcus using a murine model of pneumonia, showing considerable efficacy in reducing the bacterial load in the lungs. Lastly, we tested and observed *in vitro* efficacy of CDT with DMDC on other opportunistic pathogens, including Staphylococcus aureus, a Gram-positive commensal known to harbor extreme antibacterial resistance, *Coccidioides* spp., endemic fungi to the southwestern United States known to cause San Joaquin Valley fever (often referred to as Valley fever), and Schistosoma mansoni, the parasitic flatworm that infects over 200 million people worldwide (35, 36). Together, our data show that DMDC enhances Cu toxicity in human pathogens across three kingdoms of life (bacteria, fungi, and animals).

## RESULTS

**FIG 1:**
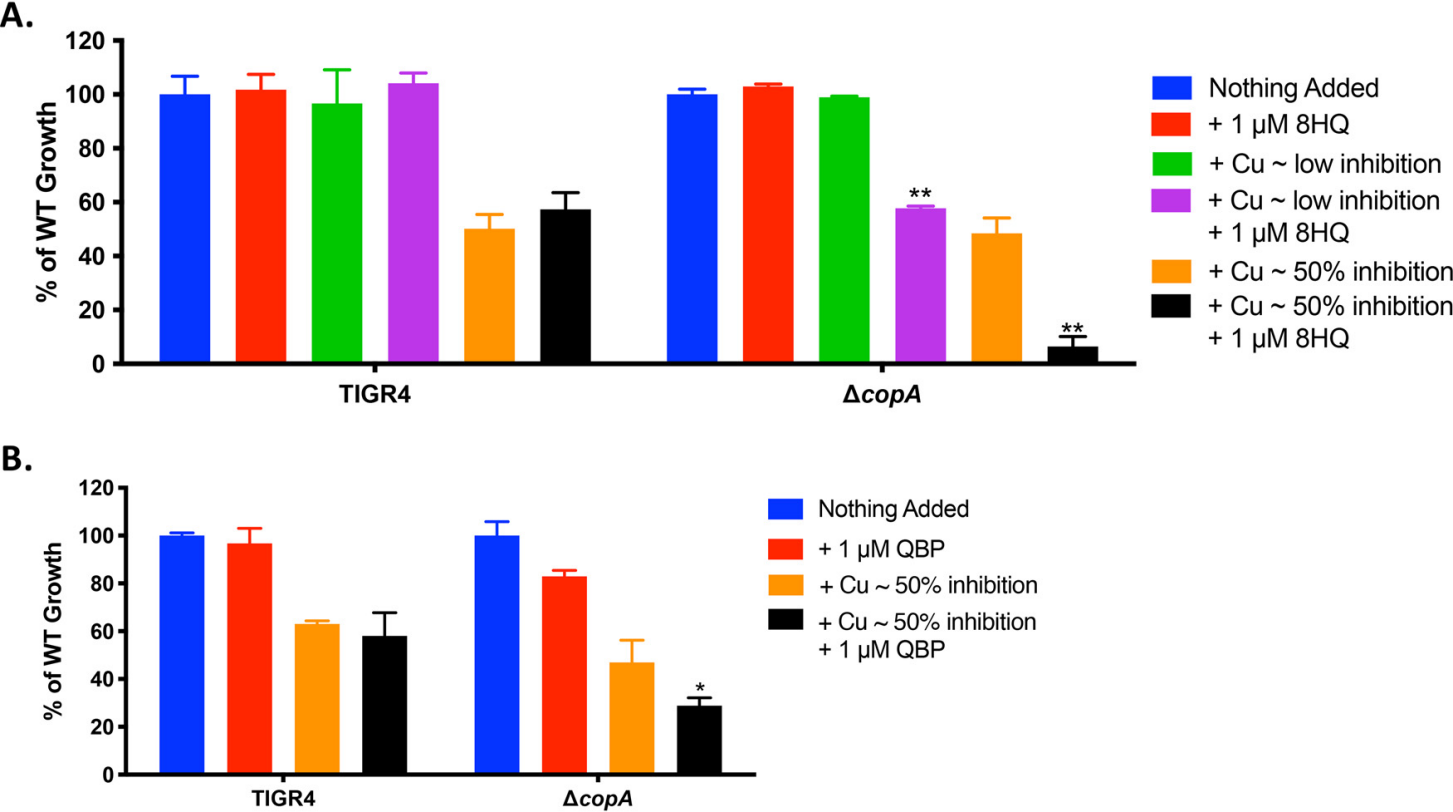
The metal-binding agent 8HQ and its prochelator form QBP do not cause a significant growth defect to WT TIGR4 but do cause a growth defect to Δ*copA* bacteria. (A) Percentage of maximal growth of WT TIGR4 or Δ*copA*-mutant bacteria under various conditions in THYB medium compared to strain growth with no copper added as measured by maximum optical density OD_600_ (maximal OD_600_ of ∼1.0). Conditions tested included no additions to THYB medium, addition of 1 μM 8HQ, addition of a low level of copper (50 μM for WT and 10 μM for Δ*copA*), addition of a low level of copper + 1 μM 8HQ, addition of a higher level of copper leading to half of maximal bacterial growth (500 μM for WT and 50 μM for Δ*copA*), and addition of a higher level of copper + 1 μM 8HQ. (B) Percentage of maximal WT TIGR4 growth under various conditions as measured by maximum optical density OD_600_. Conditions tested included no additions to THYB medium, addition of 1 μM 8HQ, addition of a low level of copper (500 μM for WT and 50 μM for Δ*copA*), addition of a low level of copper + 1 μM 8HQ, addition of a higher level of copper leading to half of maximal bacterial growth (50 μM for WT and 10 μM for Δ*copA*), and addition of a higher level of copper + 1 μM 8HQ. All bars represent mean percentage of WT growth ± standard error of the mean (SEM) with a minimum of *n* = 12 replicates per condition across 3 independent replicates. Statistical differences were measured by Student’s *t* test; *, *P* < 0.01; **, *P* < 0.001.

Experiments performed by Festa et al. demonstrate that 8HQ exhibits CDT against fungal and bacterial pathogens (21). They show experimentally that 8HQ’s activity as an ionophore increases intracellular copper, overwhelming the copper resistance mechanisms of the fungus Cryptococcus neoformans, leading to fungal killing (21). Due to its inherent toxicity in mammals, Festa et al. added an H_2_O_2_-labile pinanediol-borate group to 8HQ’s hydroxyl group, creating quinoline boronic acid pinanediol ester, or QBP, to be cleaved under conditions of elevated H_2_O_2_ levels (21). Festa et al. showed efficacy of QBP under H_2_O_2_-rich conditions and in environments such as in the phagolysosome of a macrophage, thus adding targeted efficacy. In contrast, we saw no 8HQ-mediated CDT for wild-type (WT) pneumococcus at several growth inhibitory levels of copper inhibition and thus, unsurprisingly, no CDT for QBP as well (Fig. 1A and B). However, a copper exporter-deficient mutant (Δ*copA*) (37) was susceptible to both QBP and 8HQ at all copper concentrations tested (Fig. 1A and B). The effect of QBP did not exhibit as much CDT on the Δ*copA* mutant compared to 8HQ, but it was still a significant reduction (Fig. 1A and B). These data demonstrated that CDT is possible in the pneumococcus, but only under specific conditions and potentially with different compounds.

Rather than a large-scale approach, we tested 21 compounds with known or predicted copper affinity to evaluate their level of CDT (Tables S1 and S2 in the supplemental material). These compounds were found via literature search of chelation and finding commercially available similar structures. While some compounds were already in complex with metal, such as copper phthalocyanate or sodium copper chlorophyllin, others, such as penicillamine, had been used for clinical metal chelation therapy in patients with Wilson’s disease (38). To test compounds, we used growth curves as an initial screen followed by killing curves. The growth curves were used to test if lag phase bacteria can grow under the constant stress of copper plus compound and if differences from wild type were observed; we used killing curves to examine if any demonstrated CDT from growth curves was bactericidal. Supplemental tables, as based on the growth curves, are broken into (i) compounds that had no effect (no effect) (Table S1), (ii) compounds that copper restored growth in the presence of the compound or, with high concentrations of copper, the compound rescued toxicity in WT or the Δ*copA* mutant (protective), (iii) compounds that showed a concentration-dependent effect of CDT and protection (protective synergistic switch compounds), (iv) compounds that showed CDT with just the Δ*copA* mutant (mutant synergistic compounds), and (v) compounds that showed synergism against the wild-type pneumococcus (WT synergistic) (Table S2).

**FIG 2:**
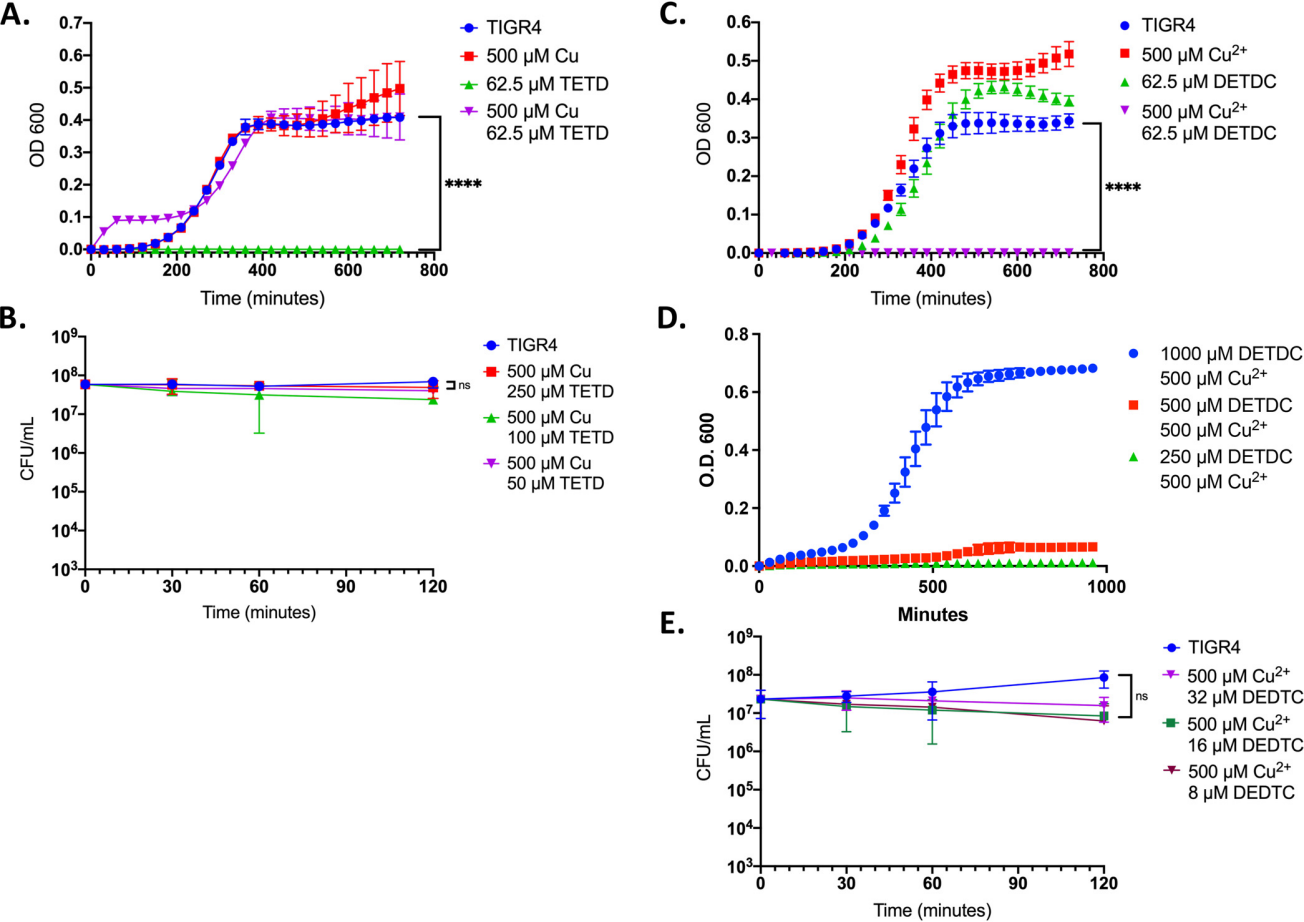
CDT not observed for disulfram (Antabuse, tetraethylthiuram disulfide [TETD]) but observed for diethyldithiocarbamate (DETDC). (A) Growth curve of WT TIGR4 exposed to indicated concentrations of copper sulfate and/or TETD. (B) Killing curve of WT TIGR4 exposed to indicated concentrations of copper sulfate and TETD. All bars represent mean ± standard deviation (SD) with *n* = 3 across 3 independent replicates. Statistical differences measured by Student’s *t* test; ****, *P* < 0.0001; ns, not significant. (C) Growth curve of WT TIGR4 exposed to indicated concentrations of copper sulfate and DETDC. (D) Growth curve of WT TIGR4 exposed to increasing concentrations of DETDC with a constant level of copper of 500 μM. (E) TIGR4 pneumococci were exposed to the indicated concentrations of copper sulfate and DETDC. All bars represent mean percentage ± SD across 3 independent replicates. Statistical differences were measured by Student’s *t* test; ****, *P* < 0.0001.

Disulfiram (Antabuse, tetraethylthiuram disulfide [TETD]) has previously been seen to have CDT against M. tuberculosis (27). Thus, we tested TETD to see if it had any CDT against the pneumococcus. We found that while TETD alone prevented growth at multiple concentrations, adding copper returned growth to wild-type levels (Fig. 2A: Fig. S1A and B). Further, we observed that TETD alone or combined with copper did not show bactericidal activity (Fig. 2B). TETD can be reduced to *N*,*N*-diethyldithiocarbamate (DETDC) within a matter of minutes inside the host (39). We found that while DETDC had CDT for concentrations ≤250 μM, much higher concentrations were ineffective for reasons unknown (Fig. 2C and D). Even so, there was no bactericidal activity measured in the killing curve, thus eliminating this compound as a viable CDT candidate moving forward (Fig. 2E).

**FIG 3:**
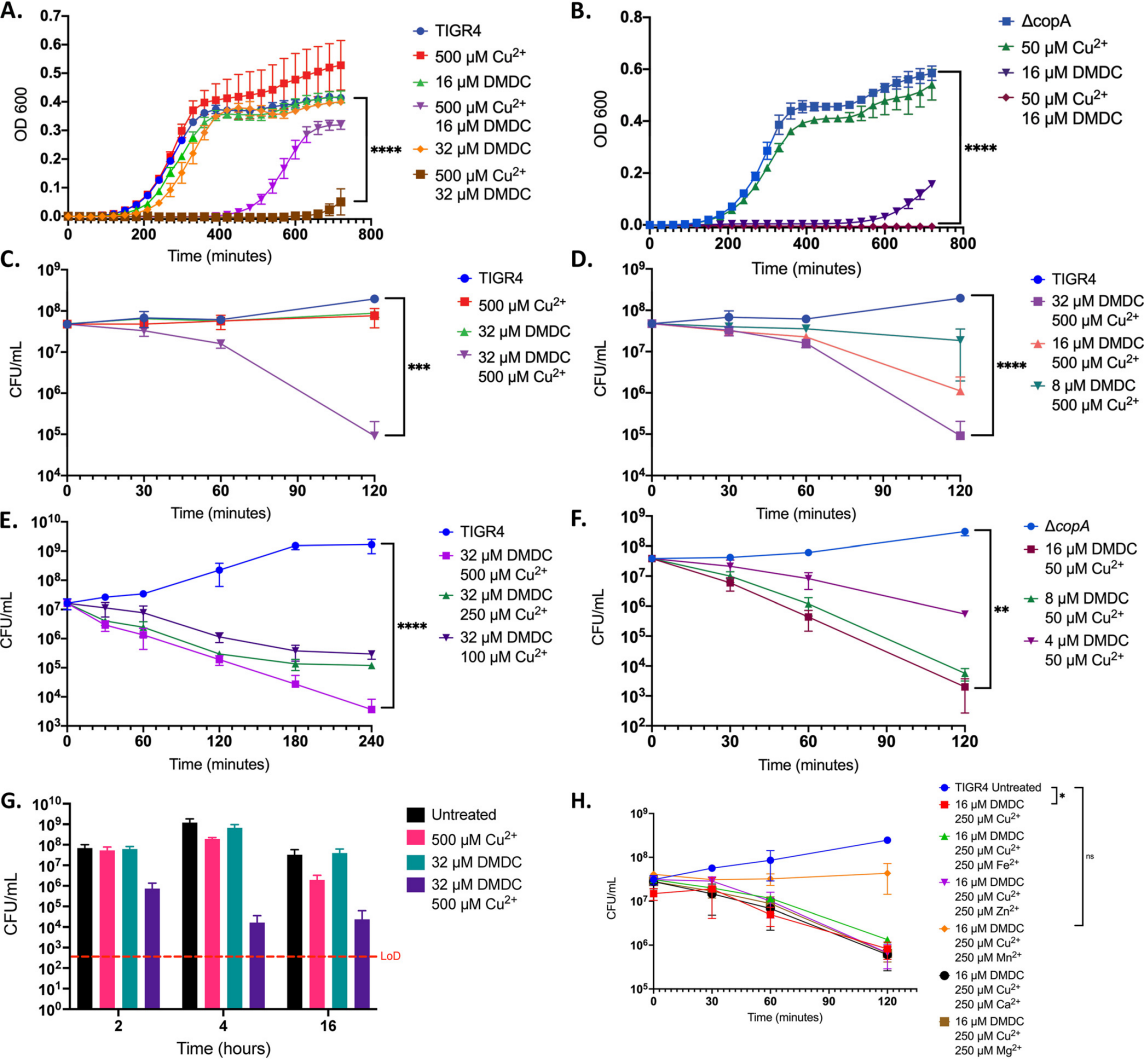
Robust bactericidal CDT observed for dimethyldithiocarbamate (DMDC). (A) Growth curve of WT TIGR4 exposed to the indicated concentrations of copper sulfate and DMDC. (B) Growth curve of the Δ*copA*-mutant strain exposed to the indicated concentrations of copper sulfate and DMDC. (C) Killing curve for WT TIGR4 bacteria exposed to the indicated concentrations of copper sulfate and DMDC, showing viable CFU over time. (D) Killing curve of WT TIGR4 bacteria with increasing concentrations of DMDC with a level of copper set to 500 μM. (E) Killing curve of WT TIGR4 bacteria with various concentrations of copper with a level of DMDC set at 32 μM over a 4-h time period. (F) Killing curve of the Δ*copA*-mutant strain exposed to the indicated concentrations of copper sulfate and DMDC. (G) Killing curve of WT TIGR4 bacteria with increasing concentrations of DMDC with a level of copper set to 500 μM at 2, 4, and 16 h. (H) Killing curve of WT TIGR4 bacteria under copper and DMDC conditions supplemented with the addition of other metals as indicated, showing that addition of 250 μM Mn^2+^ rescues CDT for 16 μM DMDC and 250 μM Cu^2+^. All bars represent mean ± SD with *n* = 3 across 4 independent replicates for growth curves and across 3 independent replicates for killing curves. Statistical differences were measured by Student’s *t* test; ****, *P* < 0.0001; ns, not significant.

*N*,*N*-dimethyldithiocarbamate (DMDC) is a compound related to DETDC with methyl groups replacing ethyl groups. We sought to examine DMDC for its ability to cause CDT *in vitro* to determine if substitutions at this position would change the effects. In growth curves, we observed CDT for DMDC in a concentration-dependent manner with both TIGR4 and the *ΔcopA* mutant (Fig. 3A and B). In the killing curve assay, we observed bactericidal activity in a DMDC-, copper-, and time-dependent manner with TIGR4 (Fig. 3C to E). DMDC also showed CDT in the killing curve with the TIGR4 *ΔcopA* mutant relative WT (Fig. 3F). To further examine the bactericidal activity of DMDC, we extended our killing curve in TIGR4 to 16 h. We found the same number of bacteria at 4 h as we did at 16 h, implying that DMDC can be broken down over time or sequestered by the bacterial milieu of live, dead, and dying bacteria (Fig. 3G).

There are currently 100 different serotypes of S. pneumoniae. Therefore, we wanted to make sure that this CDT was not specific to just TIGR4 (serotype 4). In using the same concentrations of DMDC and copper in the growth curves and the killing curves, we observed similar levels of CDT in both serotype 2 (D39) and serotype 3 pneumococcus (Fig. S2A to D).

Previously we observed that copper can displace manganese in S. pneumoniae ribonucleotide reductase NrdF and that this mismetallation could be rescued by adding excess manganese (16). Therefore, to test if DMDC was facilitating a similar method of toxicity, we added both copper and DMDC to bacteria in a killing curve and tried to rescue toxicity with manganese, zinc, iron, calcium, and magnesium. Of these metals, only manganese was able to rescue the CDT caused by DMDC (Fig. 3H). This experiment provides early evidence to the mechanism of DMDC, as it displays an ability to exacerbate copper toxicity that can be rescued with manganese supplementation.

We saw a number of compounds that had no effect against the wild-type pneumococcus, such as pyridoxine dihydrochloride (Fig. S3), but had an effect on the Δ*copA* mutant, such as 1,10-phenalthroline monohydrate (Fig. S4A and B), as well as copper-mediated protection against the compound and the protective synergistic switch. As none of these compounds displayed definitive CDT in the wild-type strain, we only continued further study of DMDC.

**FIG 4:**
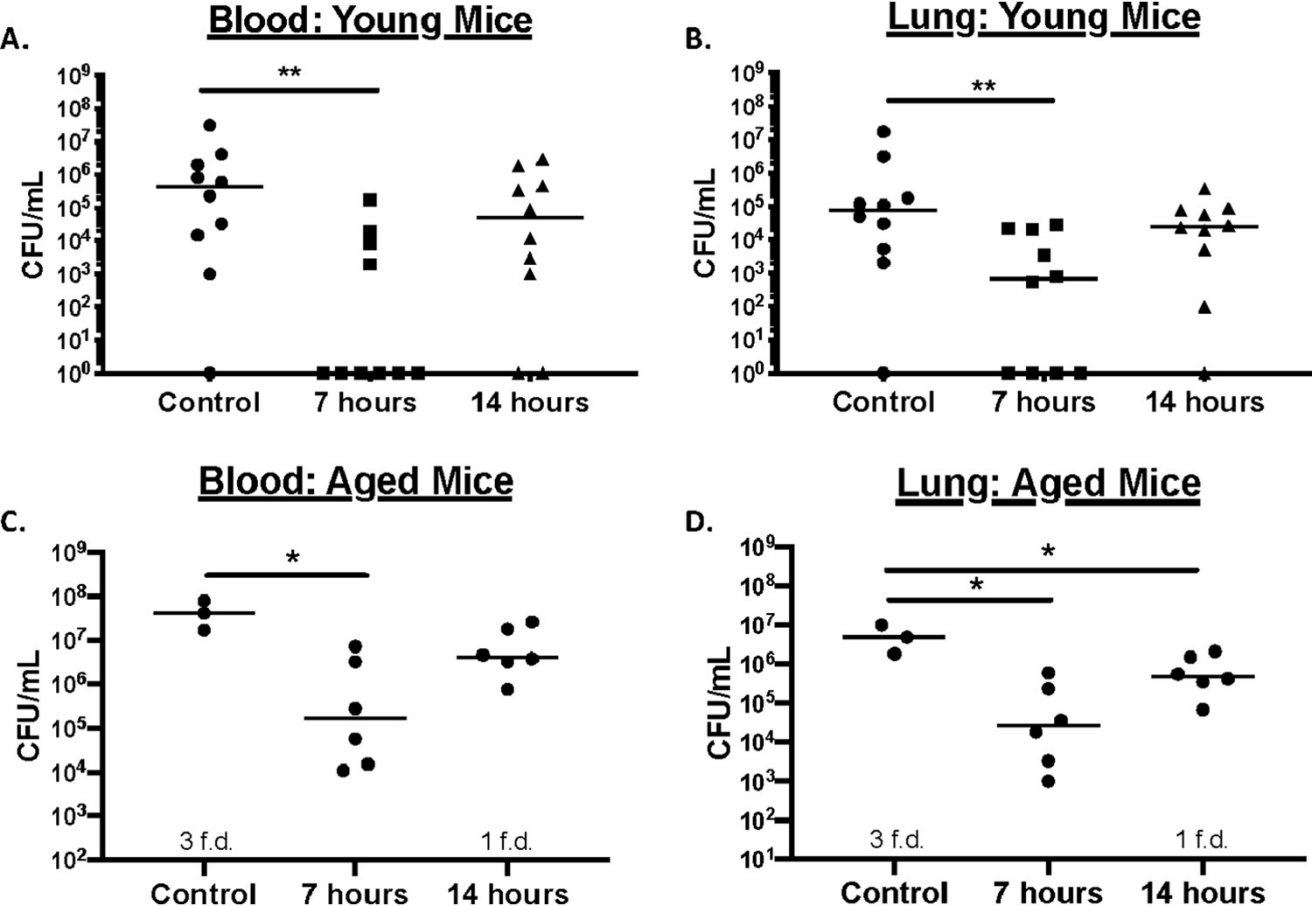
DMDC is an effective antibiotic against a murine Streptococcus pneumoniae infection model. Two groups of five 8-week-old mice (A, B) (*n* = 10) or two groups of three 18-month-old mice (C, D) (*n* = 6) per treatment were infected with bacteria at time zero and treated with DMDC at either 7 or 14 h postinfection. At 48 h postinfection, animals were sacrificed and blood (A, C) or lung titers (B, D) (*n* = 8) were measured. Mann-Whitney Wilcoxon rank sum tests were used to measure significant differences at a *P* value of <0.05 (*) and a *P* value of <0.01 (**). The bar within the data set represents the median. The term “f.d.” means “found dead.”

While the CDT efficacy of a compound against S. pneumoniae in a complex medium *in vitro* is promising, determining if DMDC works within an animal model is an important step to developing it for therapeutic use. The concentration of copper inside the infected lung is roughly 10 μM and is 40 μM in infected blood (33). Given these concentrations and the nutrient-limited environment inside the host, we decided to give DMDC at different concentrations and time points to test if bacterial burden could be reduced. The TIGR4 strain of S. pneumoniae is invasive and readily enters the bloodstream during lung infection (37). Therefore, we decided to test both lung and blood titers after 2 days postinfection. Giving a 100% lethal dose (LD_100_) to the mice intranasally, we observed a significant decrease in bacterial titers in the blood and lungs after 48 h in 8-week-old mice that were given 25 μl of 10 mM DMDC intranasally (approximately 1.6 mg/kg) 7 h after infection (Fig. 4A and B). The median titers of the 5 mM DMDC concentration and the 10 mM amount given at 14 h postinfection were lower, but the data were not significant (data not shown; Fig. 4A and B).

As the pneumococcus is also particularly detrimental to elderly human populations, we also tested the ability of DMDC to reduce bacterial burden in 18-month-old geriatric mice. Here, we not only observed significantly reduced titers in both blood and lung in the DMDC 7-h postinfection groups but also observed reduced titers in the lung 14 h postinfection (Fig. 4C and D). Taken together, these data indicated that DMDC is a viable candidate for treating pneumococcal lung infections.

We were interested in how broad spectrum DMDC could be to other pathogenic organisms. First, we tested DMDC against S. aureus. While S. aureus is a well-known cause of skin infections, toxic shock syndrome, and bacteremia, it can also cause pneumonia. 8HQ has been shown to have CDT against S. aureus (21); thus, we hypothesized that DMDC would demonstrate a similar antibiotic effect. In a growth curve, we observed that while DMDC had inherent toxicity, the addition of copper caused completely ablated bacterial growth of methicillin-sensitive and methicillin-resistant S. aureus (MSSA and MRSA, respectively) (Fig. S5A and B). However, upon performing the killing curve for 2 h, we saw no CDT for DMDC against our MSSA strain compared to the control population, copper alone, or DMDC alone (Fig. S5C). Taken together, while DMDC is effective at preventing growth at the lag phase, with higher concentrations of bacteria in a different phase of growth, it was bacteriostatic at best and had no effect at worst.

The next pathogen we tested DMDC against was the fungus *Coccidioides posadasii*. The life cycle of *Coccidioides* species is to transition from mycelia in the environment, which generates arthroconidia, and, if inhaled, grow as spherules in the lungs. Endospores develop within spherules, and, with spherule rupture, each can propagate into a new spherule to perpetuate and expand the infection. While *Coccidioides* is not spread from person to person and can be suppressed by the host immune system, severe cases require antifungals and, even so, this is sometimes not enough to clear the potentially lifelong infection (40).

**FIG 5:**
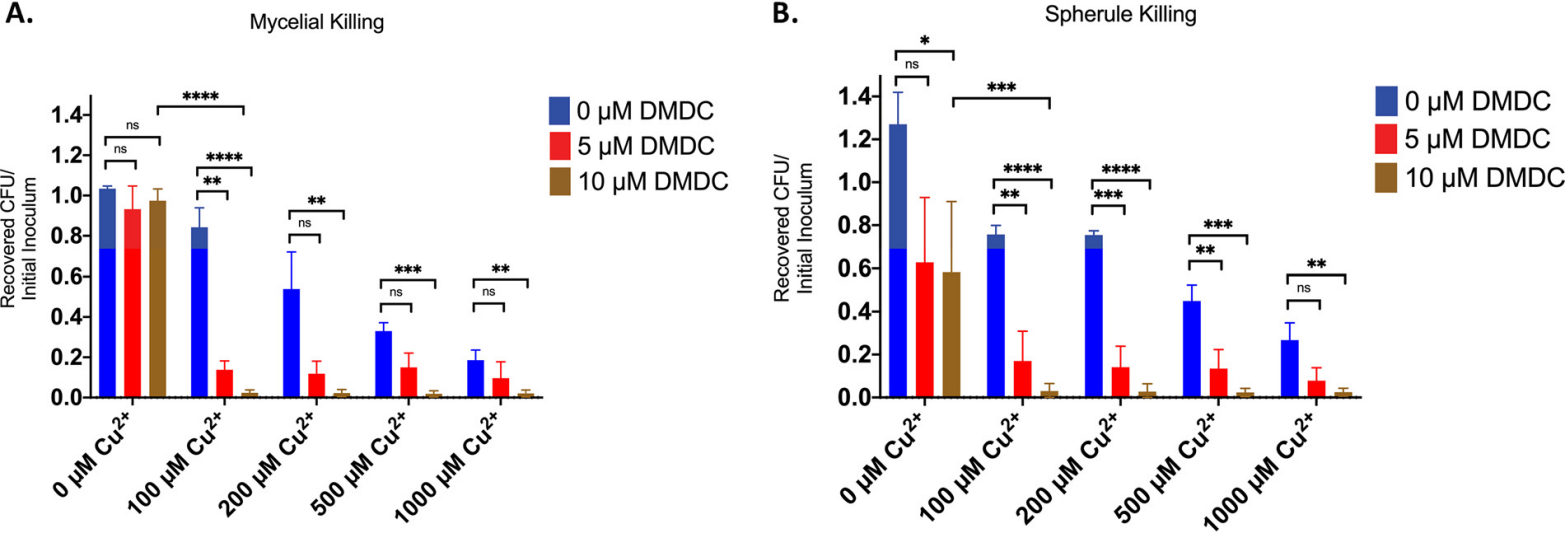
*Coccidioides posadasii* displays decreased recovery after exposure to DMDC and copper. CDT was measured in mycelial (A) or spherule (B) killing after incubation in the listed concentrations of DMDC and copper for 48 h. All bars represent mean percentage ± SEM with a minimum of *n* = 3 replicates per condition across 3 independent replicates. Statistical differences were measured by Student’s *t* test; *, *P* < 0.05; **, *P* < 0.01; ***, *P* < 0.001; ****, *P* < 0.0001; ns, not significant.

We expected that *C. posadasii* would be susceptible to DMDC, as zinc dimethyldithiocarbamate is a common fungicide. We tested DMDC for CDT against *C. posadasii* in the mycelial and spherule stages. After various concentrations of DMDC and copper (similar to bacterial killing curves) and after observing a reduction in viable *C. posadasii* organisms, we concluded that DMDC had CDT against both mycelial and spherule life stages in a copper- and DMDC-dependent manner (Fig. 5A and B). Taken together, based on these *in vitro* data, DMDC is a viable option for future therapeutic studies for *C. posadasii* and the other species of the genus, for example, *Coccidioides immitis*. After demonstrating both antibacterial and antifungal effects of DMDC and through additional collaboration, we sought to test DMDC against an animal parasite.

**FIG 6:**
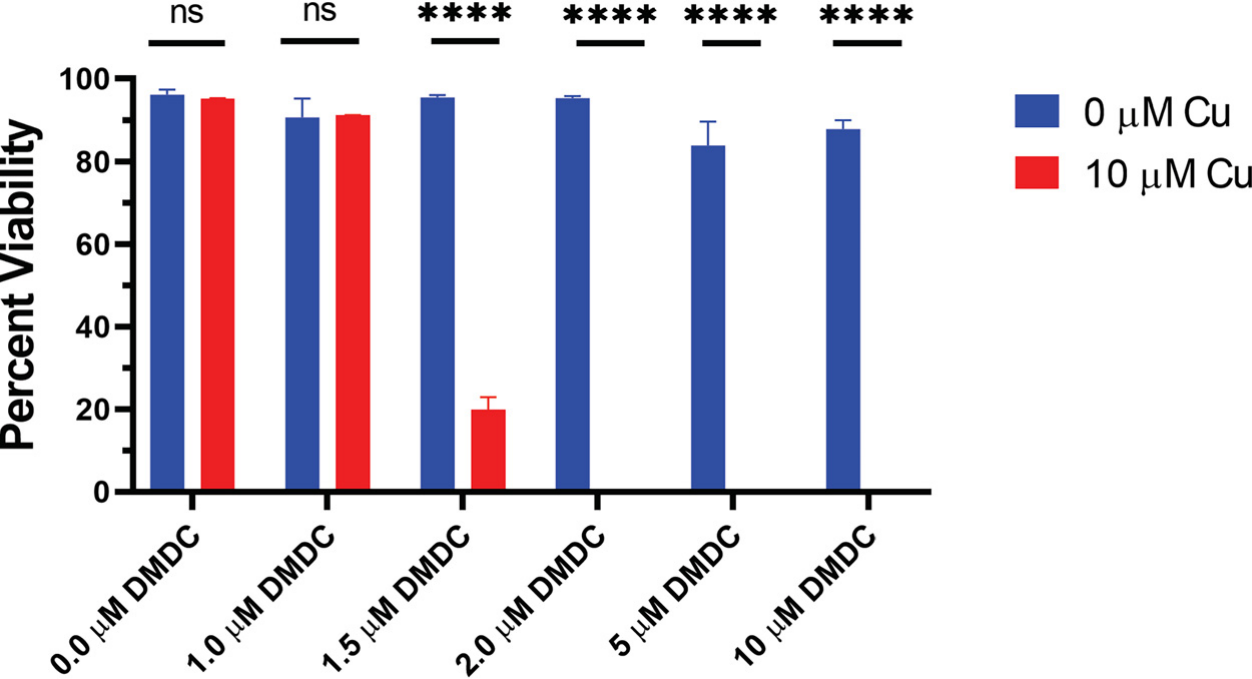
DMDC in combination with copper decreases lung-stage Schistosoma mansoni viability. DMDC was added at the indicated concentrations with (red bars) or without (blue bars) 10.0 μM CuSO_4_. Bars represent mean percentage viability ± SD with *n* = 90 per biological replicate with three independent replicates. Statistical differences were measured using multiple paired *t* tests with two-stage step-up (Benjamini, Krieger, and Yekutieli) and a false-discovery rate (FDR) of 1.00%; ****, *P* < 0.0001; ns, not significant.

*Schistosoma* life cycles require both a molluscan intermediate host and a definite mammalian host. After adhering to host skin, their larvae, called cercariae, bore through the skin of mammals using proteases. The adult worms pair and mate, producing hundreds of eggs daily. Schistosomiasis, the host’s immune response to these eggs, can lead to hepatosplenomegaly, pulmonary hypertension, urethral and bladder fibrosis, bladder and colorectal cancer, and death (41, 42). The primary treatment for schistosome infection has been praziquantel; however, its efficacy in single dosage and noncompliance as a result of its taste and gastrointestinal side effects has created challenges in treatment (43). To test the CDT against S. mansoni, we used newly transformed and lung-stage schistosomula, two developmental stages where praziquantel is not effective (44). We found DMDC to have CDT against S. mansoni in a compound-dependent manner in both stages (Fig. 6; Fig. S6A). There was no reduction in viability at 10 μM DMDC without copper; however, at concentrations as low as 2 μM DMDC with 10 μM copper, there was no viability (Fig. 6). Further, we found that DETDC was also efficacious as an antihelminthic of S. mansoni newly transformed and lung-stage schistosomula at the same concentrations but, similar to the results in the pneumococcus, was not effective at higher concentrations (Fig. S6B, C). Taken together, CDT is a feasible therapeutic for a variety of pathogenic organisms.

## DISCUSSION

**FIG 7:**
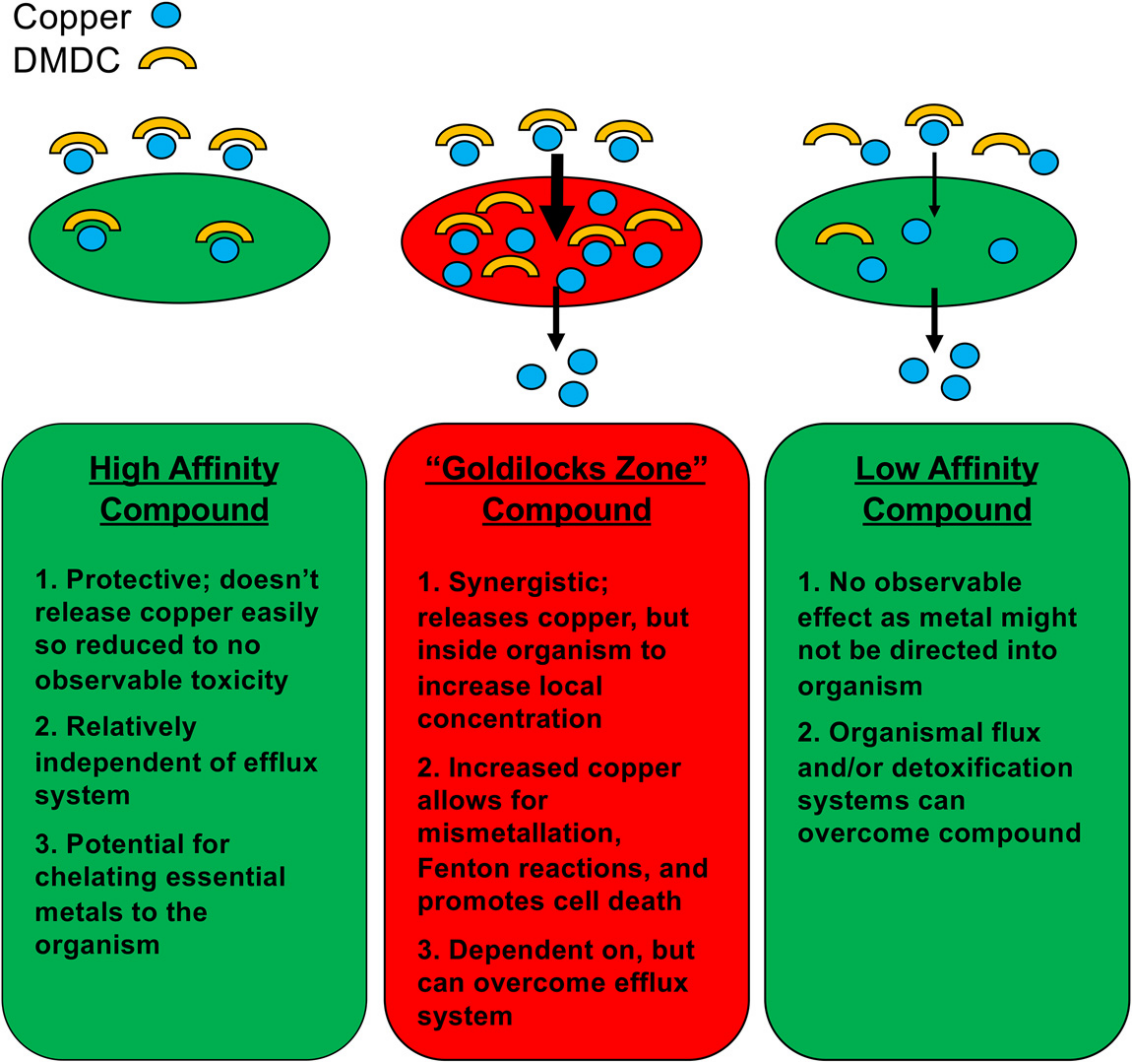
Model of DMDC CDT.

In this study, we sought to screen a variety of small molecules for bacteriostatic and bactericidal activities. We quickly realized that not all copper ionophores are created equal in this regard. Based on observing CDT in the copper exporter mutant with 8HQ but not the wild-type TIGR4 pneumococcus, we hypothesize that copper accumulation inside the bacteria impacts the effectiveness of CDT. While there are limited data describing flux of copper (specifically by comparing copper exporters), we have previously described that the Δ*copA* mutant accumulates significantly more copper than its wild-type counterpart under equal amounts of copper stress (16). Therefore, we hypothesize that the DMDC causes CDT by increasing the concentration of copper inside the pathogen (Fig. 7). This phenomenon occurs either by DMDC binding copper and increasing its import or, once inside, slowing copper’s export (Fig. 7). As such, this could be a reason why the Δ*copA* mutant was more susceptible to copper complexed with 8HQ, QBP, and DMDC (Fig. 1A, B and Fig. 3B, F).

We further hypothesize that, based on these data, ionophore affinity for copper directly affects its CDT. In essence, this screen was an exercise in finding a “Goldilocks zone” copper ionophore (Fig. 7). If the compound is bound tightly, it could be seen as protective as it strongly chelates copper from weaker ligands. Indeed, proteins that require copper generally have extremely low dissociation constants. If a compound is bound loosely to copper, it would likely have a blunted or negligible effect, especially if the organism has a robust copper efflux system. We believe this was observed with 8HQ in the wild-type pneumococcus, but, due to the reduced efficacy of the Δ*copA* mutant to export copper, we indeed observed CDT (Fig. 1A). The “just right” compound within our screen was DMDC, showing efficacy in the wild-type pneumococcus (both *in vitro* and *in vivo*), *C. posadasii*, S. mansoni, and, to a lesser degree, S. aureus. While speculative, a combination of modulating an organism’s copper flux and an ionophore’s affinity may yield a path to new treatments.

We are acutely aware that mere copper binding to an ionophore alone is not the sole factor governing CDT. Indeed, the compounds that had no effect simply may not have caused CDT due to an inability to cross the bacterial cell wall, membrane, or capsule. Descriptions of copper toxicity in biological systems tend to include either induction of increased intracellular oxidative stress via Fenton chemistry and/or mismetallation as the central components. At least in the case of the pneumococcus, our previous data show that oxidative stress did not correlate to virulence or overcoming copper toxicity (16, 37). As such, we believe that the CDT here is ultimately due to mismetallation within the organism, driven by the uncontrolled entry of DMDC-loaded copper ions (Fig. 7). This in turn leads to copper mismetallation of bacterial proteins due to its high affinity and stability for binding, resulting in bacterial death (14). While some targets of copper mismetallation have been identified (e.g., NrdF), most proteins bind tightly to and can react with copper ions (16). Exploring this perspective mechanism of toxicity will be subject to future studies.

In mouse models of infection, the concentration of copper in the infected lung and infected blood is roughly 10 μM and 40 μM, respectively (from 6 μM and 11 μM in uninfected lung or blood, respectively). One factor that likely contributes to the higher concentration of copper in these environments is the increased presence of ceruloplasmin, a copper-containing protein, as it is an acute-phase reactant that is upregulated under conditions of infection and inflammation in lungs and serum (45). While no major differences in amount produced as a function of age have been detected, the aged (65 to 95 years old) populations’ ceruloplasmin versus the 15- to 40-year-old and 40- to 65-year-old groups tend to contain copper in the oxidized form versus the reduced form (46). DMDC efficacy was significant in both the 8-week-old mice and the 18-month-old mice at the 7-h postinfection treatment but was only significant at 14 h with the geriatric mice. Differences in copper oxidation states, in complex with DMDC, and copper accessibility under treatment need to be considered regarding using DMDC as a potential future treatment for infections (Fig. 4). As such, further studies of DMDC should focus on how the compound works within a bacterium and in concert with the immune system.

DMDC appears to have some efficacy against S. aureus (Fig. S5A and B in the supplemental material); however, this was not consistent throughout our assays (Fig. S5C). While DMDC may work by a different mechanism to cause toxicity within S. aureus in comparison to S. pneumoniae, it is also conceivable that, as mentioned above, DMDC may not have met the “Goldilocks’ zone” requirement for S. aureus. Nevertheless, due to growing antibiotic resistance in S. aureus, testing if it responds to additional compounds in our screen will be subject to further study. Testing if other strains of S. aureus respond to DMDC is another avenue of future experimentation, as it is widely accepted in the S. aureus literature that there are differences between S. aureus subsp. *aureus* Rosenbach (ATCC 25923) and modern MSSA (47, 48).

DMDC appears to be a promising compound against *Coccidioides* infections, showing CDT in both the mycelial and spherule stages (Fig. 5). Coccidioidomycosis is designated by the FDA as an “orphan disease” because its prevalence is less than 200,000 infections in the United States. As such, drug development as a treatment specifically for this disease has not been pursued. In the last 4 decades, no drug has been approved by the FDA for treating coccidioidomycosis, and, currently, virtually all drug therapy for coccidioidomycosis is an off-label use of other antifungals (49). The finding here that DMDC shows promise against *Coccidioides* is particularly encouraging because of its potential for development for other infections with much larger markets. Should DMDC or a related congener be approved for any indication, postmarketing studies might result in finding a role for its treatment of coccidioidomycosis as has been the case for many other drugs.

The efficacy of DMDC in early schistosomula and DETDC in lung-stage schistosomula (Fig. 6; Fig. S6A to C) is an exciting hit for potential treatment or cotreatment of early S. mansoni. Infection clearance and reinfection are significant issues with large-scale drug administration programs (50, 51). It is likely that the variable efficacy of praziquantel against different developmental stages in the human host is exacerbating this problem. Praziquantel is highly effective against adult-stage parasites that have spent 6 weeks developing in the bloodstream but is ineffective against the early juvenile stages in the blood (44). Given that the standard method of diagnosis is egg production that only occurs when adult worms are reproducing, it is unclear if juveniles remain after treatment and a premature diagnosis of disease clearance can be given (52, 53).

Overall, we present data from a small-molecule screen showing that DMDC is a copper-dependent antibiotic against S. pneumoniae that has effectiveness against a range of pathogens, from bacteria to fungi to parasites. While choosing compounds for this study was relatively pneumococcal-centric, we acknowledge any number of the other compounds that were not effective in the pneumococcus could have had CDT to a higher degree in *C. posadasii*, S. mansoni, and S. aureus. Nevertheless, the ability of dithiocarbamates (among other copper chelators) to augment copper toxicity necessitates further examination.

## MATERIALS AND METHODS

### Bacterial culture.

Todd Hewitt broth + yeast extract (THYB) (BD Biosciences, USA) was prepared according to manufacturer’s instructions. Yeast extract was added to a final concentration of 0.2%. The final solution was set to pH 6.6. Tryptic soy agar (TSA) (Hardy Diagnostics, USA) was dissolved in deionized water and autoclaved. After cooling autoclaved TSA, 5% defibrillated sheep’s blood (HemoStat Laboratories) of final volume and 20 μg/ml neomycin were added to the solution. These plates (blood agar plates [BAP]) were used for routine culture on solid media. Copper stock solutions at 1 M were prepared from CuSO_4_ pentahydrate (VWR Life Sciences, USA) in Milli-Q-grade water (≥18.0 MΩ cm^−1^). Colonies from freshly streaked plates were placed into THYB and grown at 37°C in 5% CO_2_ to an optical density at 600 nm (OD_600_) of 0.13. To prepare working stocks of viable S. pneumoniae, growing cultures were resuspended in fresh medium and 20% (vol/vol) glycerol and were stored at −80°C. Aliquot viability and CFU were determined as discussed below before use in experiments. Glycerol stock aliquots were diluted 1:5 into THYB with indicated copper and compound concentrations for assays.

Brain heart infusion broth (BHI medium) (Sigma, USA) was prepared following the manufacturer’s instructions by dissolving in deionized water and autoclaving. Mannitol salt agar (MSA) (MilliPore Sigma, USA) was prepared following manufacturer’s instructions. MSA was dissolved in deionized water and autoclaved before pouring into petri dish plates for routine culture on solid medium. Staphylococcus aureus subsp. *aureus* Rosenbach (ATCC, 25923) (termed MSSA here) and S. aureus MRSA (ATCC, 33591) were grown at 37°C in 5% CO_2_ to an OD_600_ of 0.13 and were diluted 1:5 into BHI for assays. Aliquot viability and density were validated before use in experiments.

### Growth curves.

We purchased ≥97% pure samples of each small molecule. Each compound was dissolved in dimethyl sulfoxide (DMSO), dimethylformamide (DMF), ethanol, or water and diluted as needed. Where compounds were water soluble, dilutions were performed into sterile THYB. Clear 96-well polystyrene plates (Greiner) were arranged to test a range of concentrations from 1 mM diluted 4-fold down to 0.24 and a 0 μM control. Frozen aliquots of S. pneumoniae or S. aureus were thawed and diluted 5-fold into fresh THYB or BHI, respectively, before adding 20 μl per well into a total well volume of 200 μl (1:50 total dilution). Assay plates were loaded into a Biotek Cytation5 (Biotek, VT, USA) preequilibrated to 37°C and 4% CO_2_. Gas control settings were modified for an elevation of 720 m according to manufacturer’s directions. The protocol maintained temperature and CO_2_ while measuring absorbance at 600 nm every 30 min for 12 to 16 h.

### Killing curves.

Aliquots of S. pneumoniae and S. aureus were thawed and diluted 10-fold into assay conditions prepared in THYB or BHI, respectively. After the indicated incubations at 37°C in 5% CO_2_, samples were serially diluted, plated on BAP or MSA (respectively), incubated overnight at 37°C in 5% CO_2_, and counted to determine viable CFU. Colonies on each plate were counted and multiplied by an appropriate dilution factor based on which dilution it was to determine CFU.

### Animal experiments.

All mouse studies were conducted with prior approval and under the guidelines of the IACUC at the University of Arizona (IACUC protocol number 18–410, R35 GM128653). All mice were maintained in a biosafety level 2 (BSL2) facility and monitored daily for signs of moribund. Eight-week-old female BALB/cJ mice (Jackson Laboratory) or 18-month-old male and female C57BL/6 (National Institute on Aging) mice were anesthetized with 3% isoflurane and intranasally infected with an inoculum of 1 × 10^7^ CFU viable S. pneumoniae in 25 μl of Tris-buffered saline (TBS; 50 mM Tris, 150 mM NaCl, pH 7.4). Cohort controls were given 25 μl of TBS. At 7 or 14 h postinfection, mice were treated with doses of intranasal DMDC (0.8 mg/kg or 1.6 mg/kg) in 25 μl of TBS. Mice were sacrificed by CO_2_ asphyxiation and immediately dissected for lung and blood collection 48 h postinfection. Lung tissue was collected into 1.5-ml tubes containing 500 μl of phosphate-buffered saline (PBS; Gibco) after a brief initial wash in 500 μl of PBS to remove any excess blood during dissection. The tissue was then homogenized and centrifuged for 30 s at 400 × *g*. Blood samples (5-μl volume) were placed in a 45-μl volume PBS solution with heparin (10 UI/ml). Both lung and blood samples were then serially diluted 1:10 and plated on TSA blood plates and incubated overnight at 37°C and 5% CO_2_ for growth. Resulting bacterial colonies were counted for quantification and comparison.

### *Coccidioides posadasii* viability studies.

*Coccidioides posadasii* strain Silveira cultures (Cp) were grown to maturity on 2× glucose-yeast extract (GYE) agar, and arthroconidia (spores) were harvested as previously described in reference 54. Cultures were then incubated with the indicated concentrations of CuSO_4_ and sodium dimethyldithiocarbamate. The mycelial phase test was performed at the mycelial phase for 48 h at 37°C, static. The spherule test was performed at spherule phase for 72 h at 38°C, 180 rpm, and 20% CO_2_ in modified converse medium (55, 56). After the respective incubation periods, each sample was diluted 1:100 and plated on GYE to measure viability. The GYE plates were incubated at 37°C for 4 to 7 days. All manipulation of live fungus was performed at biosafety level 3 with University of Arizona Institutional Biosafety Committee approval.

### Juvenile parasite collection and culture.

*Biomphalaria glabrata* snails infected with Schistosoma mansoni (Naval Medical Research Institute [NMRI] strain) were obtained from the Biomedical Research Institute (BRI; Rockville, MD). Cercariae were shed, and mechanical transformation was performed as previously described (57). Newly transformed schistosomula (NTS) were cultured at 37°C in 5% CO_2_ using Dulbecco’s modified Eagle medium (DMEM; Gibco) supplemented with 10% fetal bovine serum (FBS) and 20,000 U of penicillin and 20 mg of streptomycin per milliliter (2× PenStrep).

### DMDC juvenile parasite viability screening.

Approximately 90 schistosomula per well were cultured in a 96-well plate, and each treatment was administered in triplicate. All treatments, including the untreated control samples, were performed in a 200-μl total volume of complete DMEM (Gibco) supplemented with 10% FBS and 2× PenStrep and were performed overnight. Newly transformed schistosomula were treated immediately after transformation; lung-stage schistosomula were cultured for 14 days before treatment was administered. Blood supplementation, 2.0 μl of concentrated human red blood cells with EDTA, was given after 2 days of culture for lung-stage schistosomula and was repeated every 2 days. Propidium iodide (PI) at 2 μg/ml was used to stain dead cells within the schistosomula. Fluorescein diacetate at 0.5 μg/ml was used to stain live schistosomula (58). A Leica DMI8 fluorescence microscope ×10 objective was used to observe and count individuals under brightfield using the Texas Red cube set for PI visualization. A fluorescein isothiocyanate cube set was used for fluorescein diacetate visualization. Parasites showing any level of PI staining were considered nonviable. Viable individuals were both positive for fluorescein diacetate staining and negative for PI staining. The following equation was used to calculate viability: fluorescein diacetate-positive only/well total population × 100 = viability percentage.
