# Supplementary material for: Demonstration of N,N-Dimethyldithiocarbamate as a Copper-Dependent Antibiotic against Multiple Upper Respiratory Tract Pathogens
Source: Microbiol Spectr. 2021 Sep 1;9(2):e00778-21. doi: 10.1128/Spectrum.00778-21 (PMC8557878; doi:10.1128/Spectrum.00778-21)

Supplemental Material:

Demonstration of N,N-dimethyldithiocarbamate as a copper-dependent antibiotic against multiple upper-respiratory tract pathogens

Sanjay V. Menghani[1*], Angela Rivera[1,2,3*], Miranda Neubert[1], James R. Hagerty[4], Lourdes Lewis[5], John N. Galgiani[5,6], Emmitt R. Jolly[4,7], Joseph W. Alvin[1], and Michael D. L. Johnson[1,5,6]

[1]Department of Immunobiology
University of Arizona
Tucson, AZ
[2]Department of Chemistry and Biochemistry
University of Arizona
Tucson, AZ
[3]Current affiliation: Department of Pharmacology & Cancer Biology
Duke University School of Medicine
Durham, NC
[4]Department of Biology
Case Western Reserve University
Cleveland, OH
[5]Valley Fever Center for Excellence and Department of Medicine
University of Arizona
Tucson, AZ
[6]BIO5 Institute
University of Arizona
Tucson, AZ
[7]Center for Global Health and Disease,
Case Western Reserve University
Cleveland OH

Corresponding Author: Michael D. L. Johnson
University of Arizona
1656 E. Mabel St. / P.O. Box 245221 / MRB 213 (office)
Tucson, AZ 85724
Tel: 520-626-3779 / Fax: 520-626-2100
Mdljohnson@arizona.edu

Supplemental Tables

Table 1: Structures of compounds within drug screen showing no effect on growth of WT TIGR4 bacteria as measured by OD600

Table 2: Structures of compounds within drug screen showing a significant effect on growth of WT TIGR4 or ΔcopA mutant bacteria as measured by OD600

Compounds in the “Protective” group caused a significant growth increase in the WT TIGR4 bacteria without the addition of copper. Compounds in the “Protective Synergistic Switch” group were protective for growth in certain conditions of compound concentration + Cu2+ concentration and caused growth defect in certain conditions of compound concentration + Cu2+ concentration. Compounds in the “Mutant Synergistic” group displayed CDT against the ΔcopA mutant. The compound identified in the “WT Synergistic” section, N,N-dimethyldithiocarbamate (DMDC), displays CDT. Supplemental

Figure Legends:

Supplemental Figure 1: Growth curves for representative high and low concentrations of TETD ± Copper. Growth curves of WT TIGR4 exposed to indicated concentrations of copper sulfate and TETD. Low concentration of TETD (15.6 µM) with supplementation of 500 µM $^{Cu2+}$ improves growth in comparison to the no copper condition. High concentration of TETD (250 µM) alone ablated growth while addition of 500 µM Cu^{2+} to high TETD leads to normal growth. All bars represent mean percentage ± SD with n = 3 across 3 independent replicates.

Supplemental Figure 2: CDT observed for DMDC against multiple strains of *S. pneumoniae*. (A) Growth curve of WT D39 strain exposed to indicated concentrations of copper sulfate and DMDC. (B) Killing curve for WT D39 bacteria exposed to indicated concentrations of copper sulfate and DMDC, showing viable CFU over time. (C) Growth curve of

WT Type 3 (ATCC® 6303™) bacteria exposed to indicated concentrations of copper sulfate and DMDC. (D) Killing curve of WT Type 3 (ATCC® 6303™) bacteria exposed to indicated concentrations of copper sulfate and DMDC. All bars represent mean ± SD with n = 3 across 3 independent replicates for both growth and killing curves. Statistical difference measured by Student's *t* test (****p < 0.0001).

Supplemental Figure 3: Growth curve for pyrithioxin dihydroxychloride, a representative compound with no effect on WT growth. Growth curve of WT TIGR4 exposed to indicated concentrations of copper sulfate and pyrithioxin dihydroxychloride. All bars represent mean ± SD with n = 3 across 3 independent replicates. Statistical difference measured by Student's *t* test (no significant difference calculated).

Supplemental Figure 4: Growth curve for 1,10-phenanthroline monohydrate, a representative compound with no effect on WT growth but a significant ablation of ΔcopA mutant growth. (A) Growth curve of WT TIGR4 exposed to indicated concentrations of copper sulfate and 1,10-phenanthroline monohydrate (B) Growth curve of the Δ*copA* mutant strain exposed to indicated concentrations of copper sulfate and 1,10-phenanthroline monohydrate. All bars represent mean ± SD with n = 3 across 3 independent replicates. Statistical difference measured by Student's *t* test (****p < 0.0001).

Supplemental Figure 5: Antimicrobial activity of dimethyldithiocarbamate (DMDC) against *Staphylococcus aureus*. (A) Growth curve of *S. aureus* in BHI media with indicated concentrations of copper sulfate and DMDC. (B) Kill curve of *S. aureus* exposed to indicated concentrations of copper sulfate and DMDC. All bars for growth curve represent mean percentage ± SEM with a minimum of n = 18 replicates per condition across 3 independent replicates. Statistical significance determined by the Student's t-test (****p < 0.0001).

Supplemental Figure 6. DMDC and DETDC DCT in *S. mansoni* (A) DMDC treatment was given with copper supplementation at 10 µM to newly transformed *S. mansoni* schistosomula. B. DETDC treatment was given with copper supplementation at 10 µM in the (B) lung stage or (C) Newly transformed stage. Bars represent mean percentage viability ± SD with n=90 per biological replicate with three independent replicates. Statistical differences measured using one-way ANOVA with Tukey’s multiple comparisons (****p <0.0001, ns= not significant).

Tables

**Table 1: No Effect Compounds**				
**D-penicillamine**<br>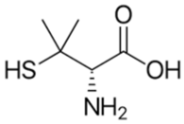	**Captopril**<br>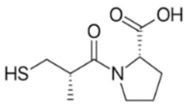	**2-aminopyridine**<br>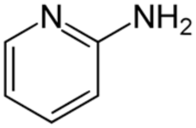	**2-thiouracil**<br>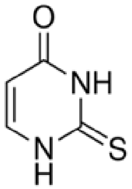	**Pyrithioxin dihydrochloride**<br>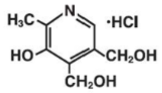
**2,3 dihydroxybenzoic acid**<br>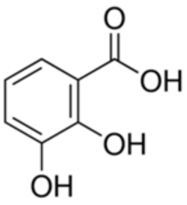	**Gallic acid**<br>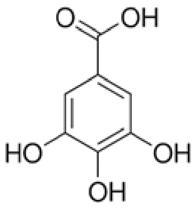	**8-quinolinesulfonyl chloride**<br>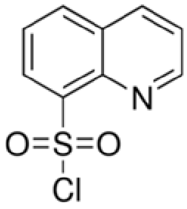	**Dicyclohexano-18-crown-6-ether**<br>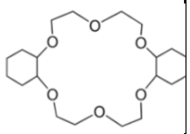	**Dibenzo-24-crown-8-ether**<br>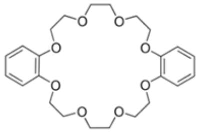
**Copper (II) phthalocyanine, beta form**<br>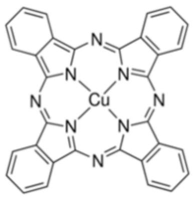	**Protoporphyrin 9**<br>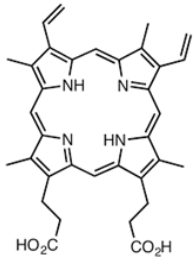	**5,10,15,20 tetrakis (4 methoxyphenyl) porphyrin**<br>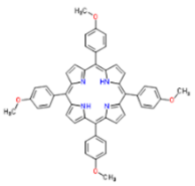	**Sodium copper chlorophyllin**<br>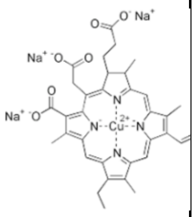	

<table>
<tr><th colspan="4">Table 2: Effective Compounds</th></tr>
<tr><th colspan="2">Protective Compounds</th><th colspan="2">Protective Synergistic Switch Compounds</th></tr>
<tr><td>2,6 pyridinecarboxylic acid
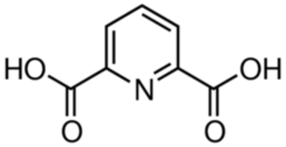</td><td>Tetraethylthiuram disulfide
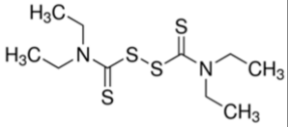</td><td>2-mercaptopyridine-n-oxide
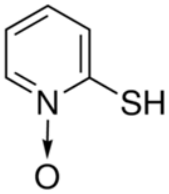</td><td>Sodium diethyldithio-carbamate trihydrate
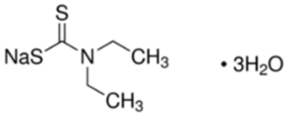</td></tr>
<tr><th colspan="3">Mutant Synergistic Compounds</th><th>WT Synergistic</th></tr>
<tr><td>1,10 phenanthroline monohydrate
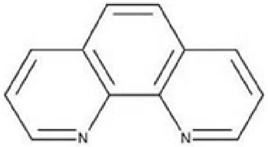</td><td>8 hydroxyquinoline
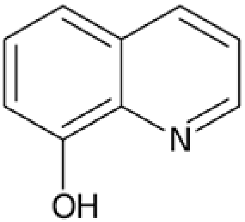</td><td>Tetrabutylthiuram disulfide
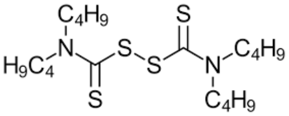</td><td>Sodium dimethyldithio-carbamate dihydrate
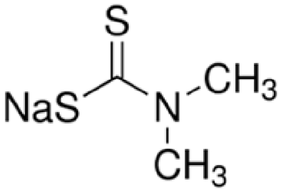</td></tr>
</table>

Supplemental Figure 1

A.

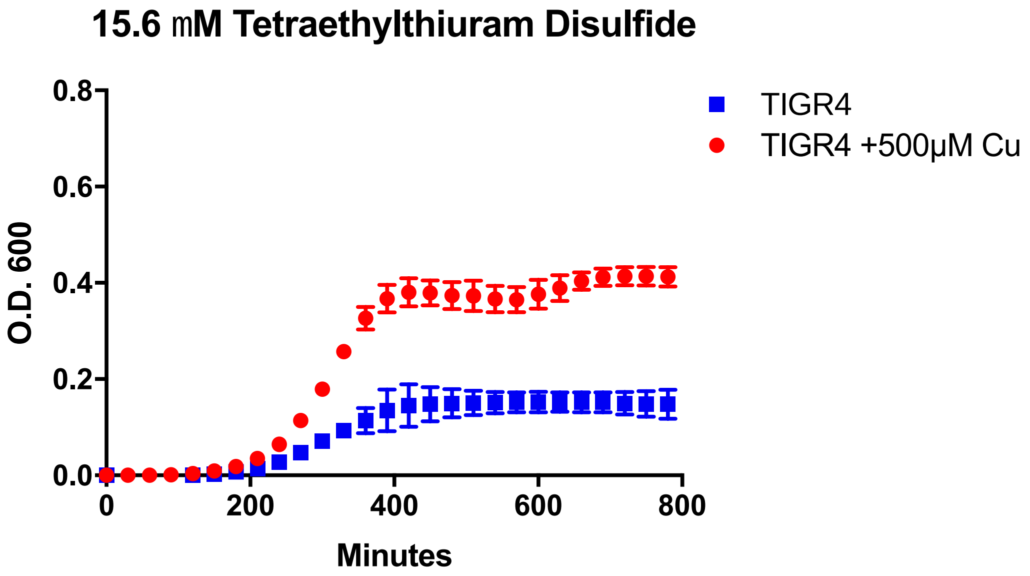

B.

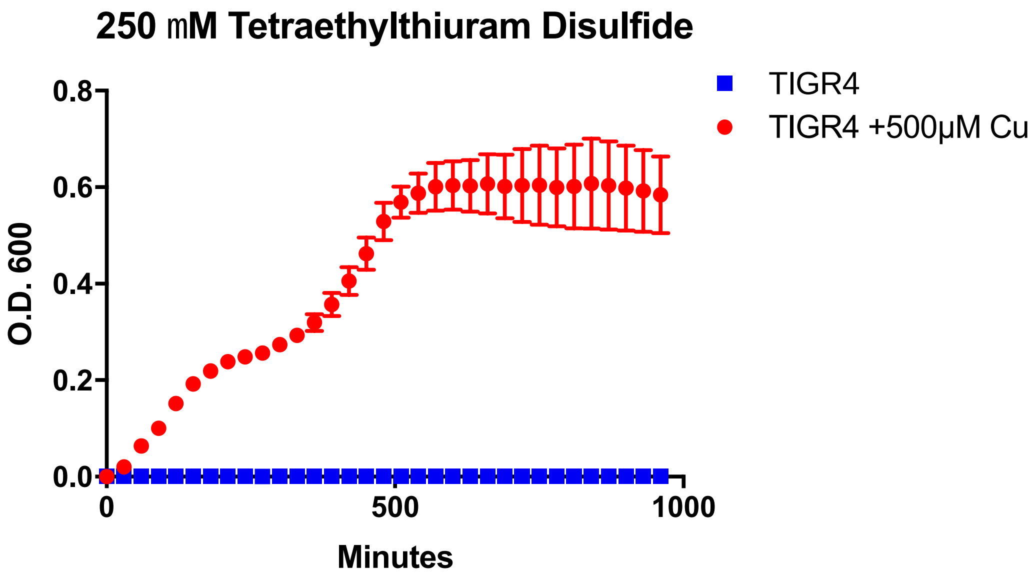

Supplemental Figure 2

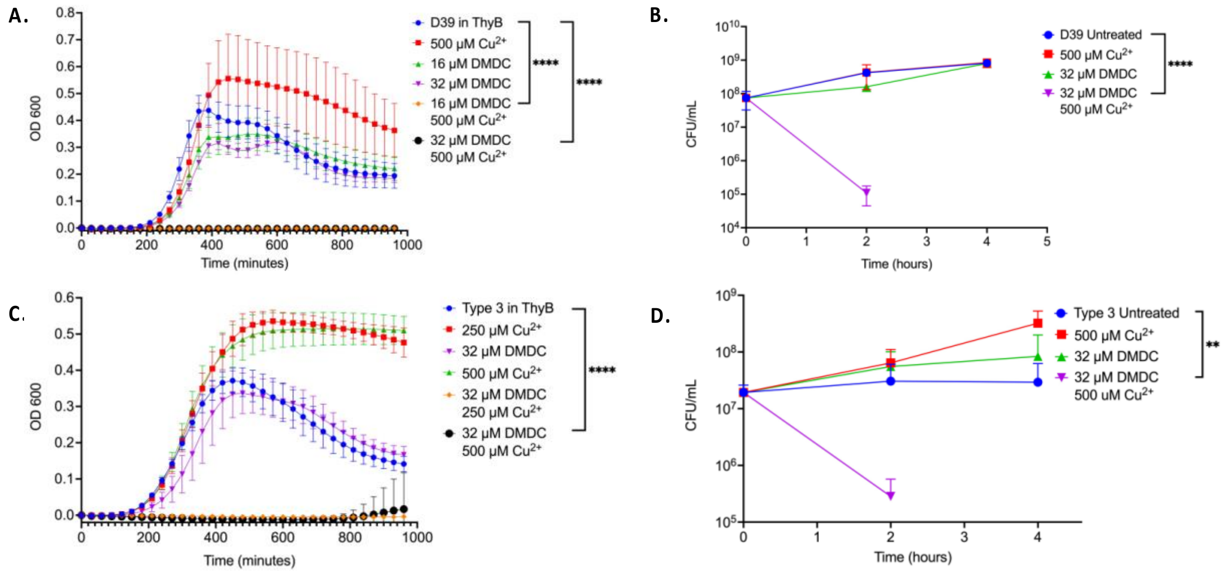

Supplemental Figure 3

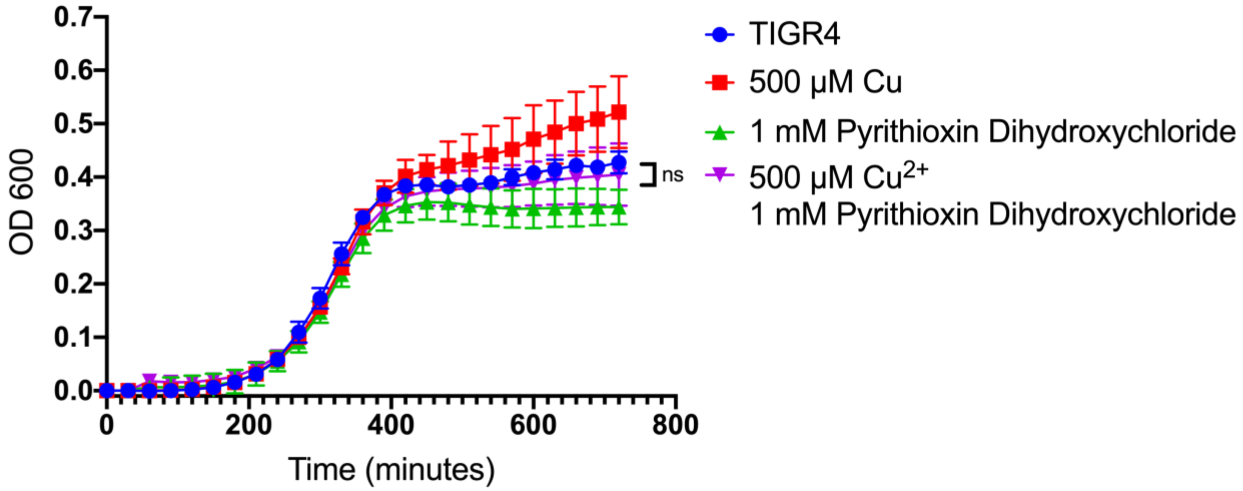

Supplemental Figure 4

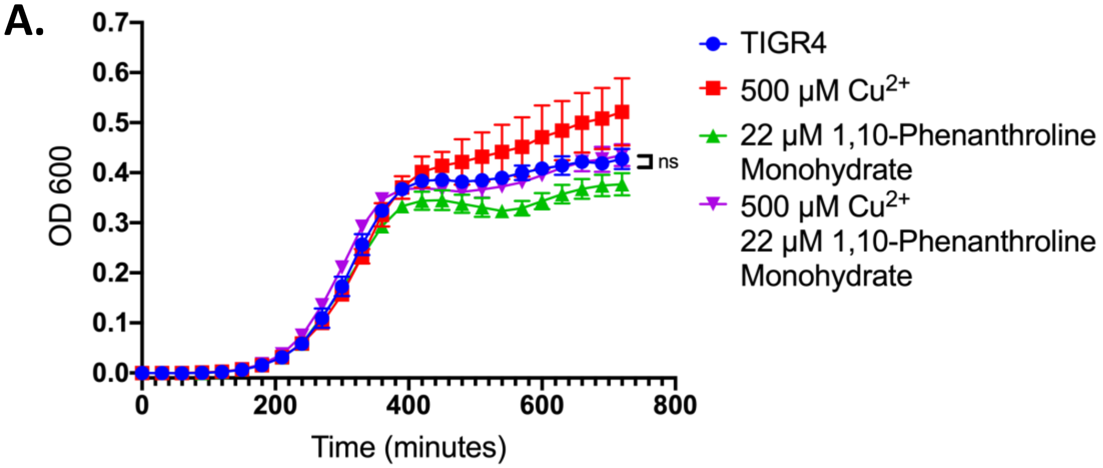

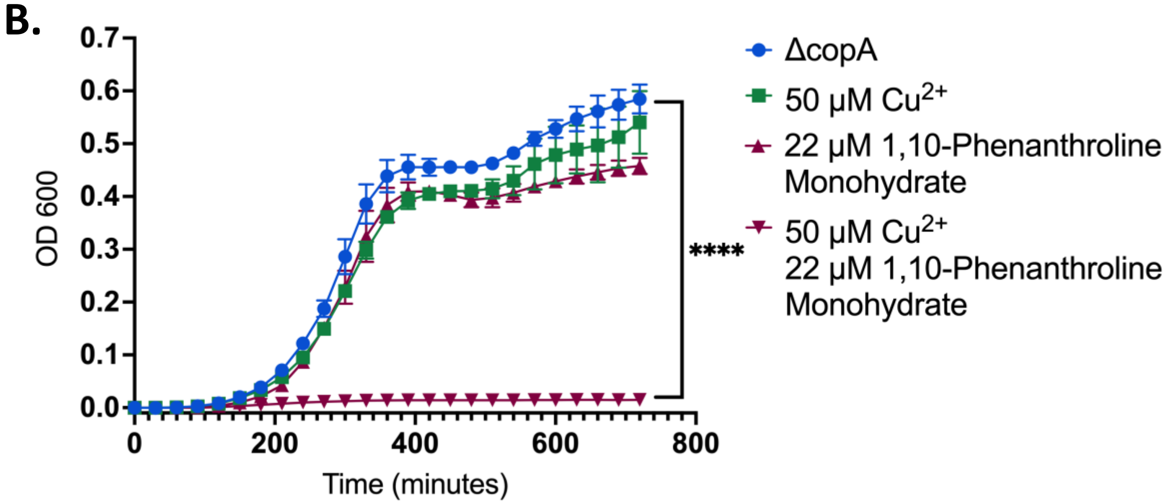

Supplemental Figure 5

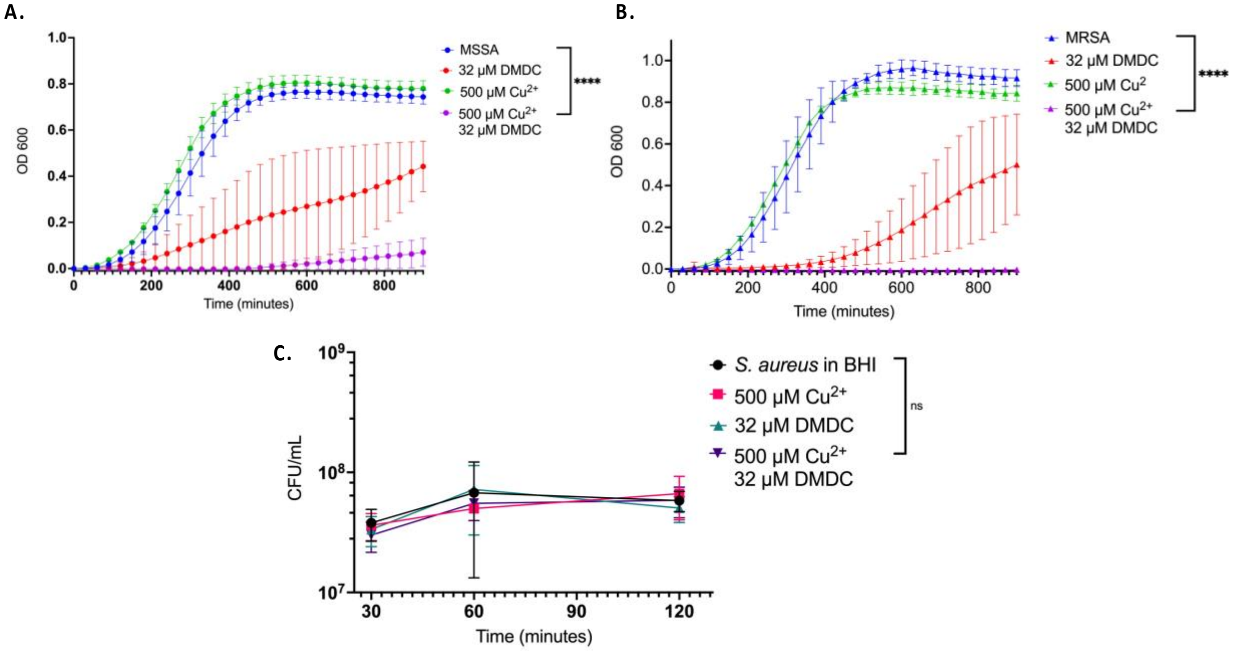

Supplemental Figure 6

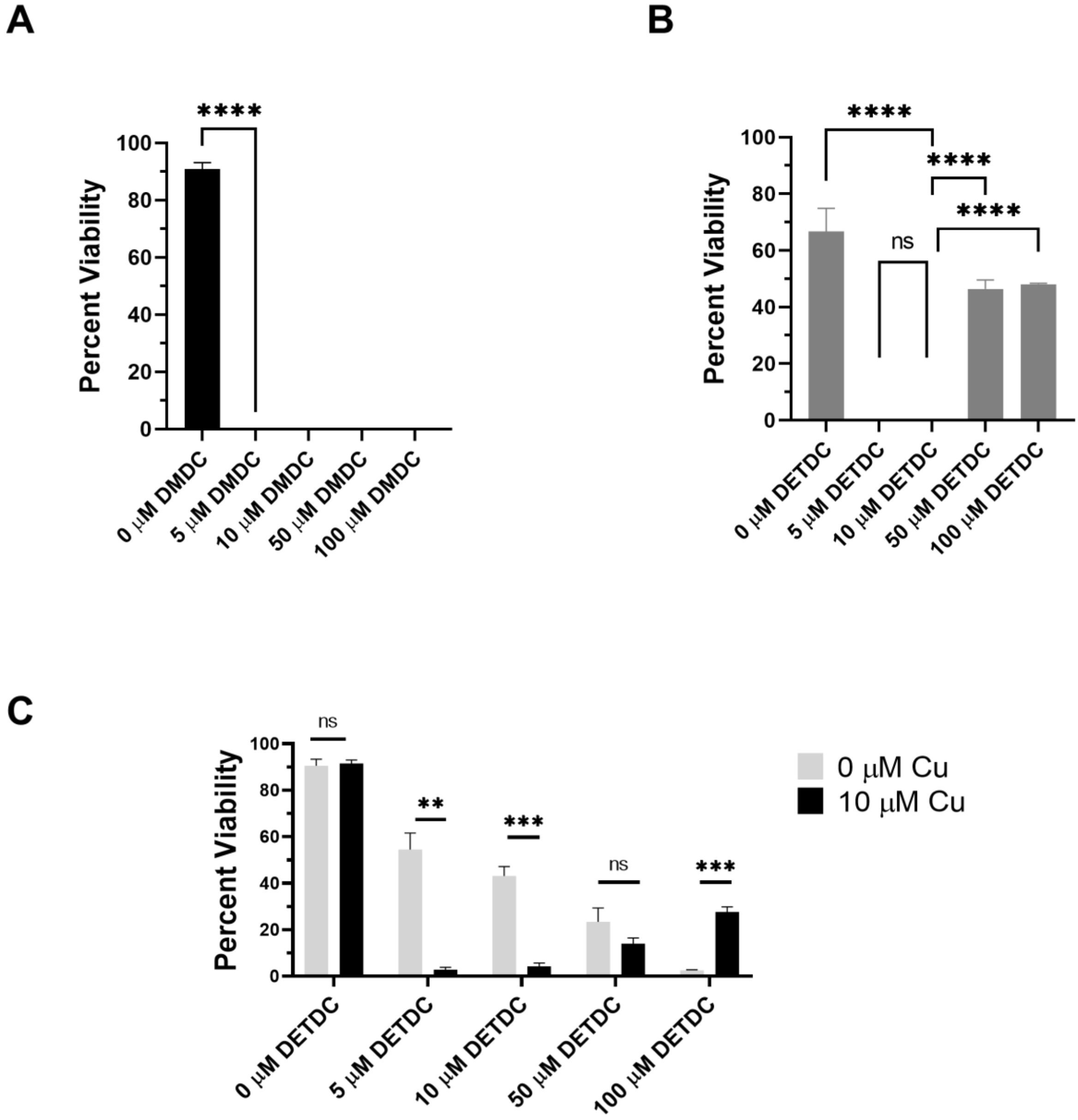

Supplement: SUPPLEMENTAL FILE 1 — Supplemental material. Download Spectrum.00778-21-s0001.pdf, PDF file, 1 MB. [file spectrum.00778-21-s0001.pdf]
